# Multi-segmented feature coupling for jointly reconstructing initial pressure and speed of sound in photoacoustic computed tomography

**DOI:** 10.1117/1.JBO.27.7.076001

**Published:** 2022-07-01

**Authors:** Kexin Deng, Xuanhao Wang, Chuangjian Cai, Manxiu Cui, Hongzhi Zuo, Jianwen Luo, Cheng Ma

**Affiliations:** a Tsinghua University, School of Medicine, Department of Biomedical Engineering, Beijing, China; b Tsinghua University, Department of Electronic Engineering, Beijing, China; c Tsinghua University, Institute for Precision Healthcare, Beijing, China

**Keywords:** photoacoustics, imaging, velocity, medical imaging

## Abstract

**Significance:**

Photoacoustic computed tomography (PACT) is a fast-growing imaging modality. In PACT, the image quality is degraded due to the unknown distribution of the speed of sound (SoS). Emerging initial pressure (IP) and SoS joint-reconstruction methods promise reduced artifacts in PACT. However, previous joint-reconstruction methods have some deficiencies. A more effective method has promising prospects in preclinical applications.

**Aim:**

We propose a multi-segmented feature coupling (MSFC) method for SoS-IP joint reconstruction in PACT.

**Approach:**

In the proposed method, the ultrasound detectors were divided into multiple sub-arrays with each sub-array and its opposite counterpart considered to be a pair. The delay and sum algorithm was then used to reconstruct two images based on a subarray pair and estimated a direction-specific SoS, based on image correlation and the orientation of the subarrays. Once the data generated by all pairs of subarrays were processed, an image that was optimized in terms of minimal feature splitting in all directions was generated. Further, based on the direction-specific SoS, a model-based method was used to directly reconstruct the SoS distribution.

**Results:**

Both phantom and animal experiments demonstrated feasibility and showed promising results compared with conventional methods, with less splitting and blurring and fewer distortions.

**Conclusions:**

The developed MSFC method shows promising results for both IP and SoS reconstruction. The MSFC method will help to optimize the image quality of PACT in clinical applications.

## 1 Introduction

Photoacoustic computed tomography (PACT, or optoacoustic computed tomography) is a hybrid imaging modality with optical absorption contrast and ultrasonic penetration depth.^1^ In most conventional optical imaging modalities, only photons within the ballistic regime are used for image formation. In PACT, however, the energy of both ballistic and diffused photons, absorbed by chromophores at a depth of up to several centimeters, is utilized for image formation.^2^^,^^3^ Fast energy deposition in the tissue causes thermo-elastic expansion and generates ultrasound emission. Therefore, the penetration depth of PACT can approach that of ultrasonic imaging (more than 5 cm), which is much deeper than most optical imaging modalities.^4^

The delay and sum (DAS) algorithm is a widely-used reconstruction method for both ultrasonic and photoacoustic imaging.^5^ This method requires an estimation of the time of flight (ToF) of the ultrasonic signal, which typically assumes the speed of sound (SoS) to be known *a priori*. In its simplest implementation, DAS assumes a single SoS for both the biological tissue and the surrounding acoustic-coupling medium (i.e., water in most cases for PACT). Alternatively, one can assign two SoS values to the tissue and its surrounding medium,^6^ which can be regarded as the first-order correction in which the tissue is assumed to be acoustically homogeneous.^7^^,^^8^ However, considering that the SoS inside soft tissues varies from 1350 (fat) to 1700  m/s (skin),^9^^,^^10^ such an assumption can sometimes be too rough and can consequently cause splitting, blurring, and distortions of structural features, thus impeding many kinds of quantification tasks.^11^^,^^12^

Studies have shown that, by integrating ultrasound tomography, the acoustic properties in the imaged field of view, including the SoS and acoustic attenuation, can be reconstructed at the expense of system complexity.^13^^–^^15^ Another type of approach employs a model-based procedure to jointly reconstruct (JR) the acoustic properties and the photoacoustic IP by iteratively updating the numerical model to match the experimental data.^16^^–^^21^ Huang et al. proved that the linearized JR problem is highly ill-conditioned and thus numerically unstable. In practice, the detection angle, spatial sampling frequency, and temporal bandwidth of the ultrasound sensors are limited, posing a greater challenge for the real-world application of the model-based JR methods.^22^^,^^23^

Inspired by adaptive optics in pure optical imaging,^24^^–^^33^ Cui et al. proposed that the wavefront distortions of PA signals caused by tissue acoustic inhomogeneity could be estimated in the frequency domain and corrected.^34^ The SoS map is estimated subsequently according to the estimated wavefronts. However, this method is based on the assumption that the acoustic attenuation is low and the signal amplitudes received by opposite detectors are comparative. For *in vivo* applications in which the above assumption is unsatisfied, the algorithm may generate erroneous estimations.

Cai et al. proposed an iterative feature coupling (FC) method to simultaneously image the IP and SoS distribution, without introducing any hardware for ultrasound tomography or implementing model-based reconstruction.^35^ In this method, a full ring detector array was separated into two 180 degree halves. The data from each half of the array was used to reconstruct a PA image, and then the SoS distribution was adjusted through iterations to maximize the correlation between the two images. The FC method can improve image quality with moderate computational cost and can simultaneously acquire a rough estimation of the SoS distribution. A potential problem of the FC method is that, because image brightness varies spatially, the reconstruction result can be overwhelmingly determined by a few bright features. Consequently, splitting, blurring, and distortions of dim image features are likely ignored by the algorithm. Moreover, this method updated the ToF map for every iteration, making it time-consuming when the degrees of freedom are high.

Here, we propose and demonstrate an advanced version of the FC reconstruction, termed multi-segmented feature coupling (MSFC), which addresses the above-mentioned problems and improves the performance of the method in terms of accuracy and speed. Instead of dividing the full ring into two halves, we segment the array evenly into eight groups. Two groups in opposite positions are considered to be a pair. For each pair, the basic feature coupling method is applied to decide a direction-specific SoS. To reduce the computational time, the distance over which PA waves propagate (in both tissue and water) is calculated and stored in a length of flight (LoF) map. All ToF values are calculated according to the LoF map instead of by the fast marching method used in the conventional FC method. Performing FC on the images reconstructed by a pair of sensor groups determines the mean SoS along the direction of the selected pair. Numerical, phantom, and *in vivo* experiments show that, with MSFC, PACT image quality improves. Furthermore, the calculated mean SoS along different directions can be used to estimate the distribution of SoS.

## 2 Method

In PACT, the PA pressure, p(r,t), is detected by a sensing element placed at r as a function of time t. In PA image reconstruction, p(r,t) is used to calculate the IP $p(r^{\prime },0)$, where $r^{\prime }$ labels the coordinates of the image. A commonly used method for PACT image reconstruction is filtered back-projection.^5^ For acoustically heterogeneous media, the formula of back-projection is

p(r′,0)=∫Ω0(2p(r,t)−2t∂p(r,t))∂t)|t=TOF^(r′,r)dΩ/Ω0, (1)

in which ${d\Omega }_{0}$ is the solid angle of the sensing element with respect to the reconstructed point $\left(r^{\prime }\right)$ and TOF^(r′,r) is the estimated ToF from r to $r^{\prime }$.

**Fig. 1:**
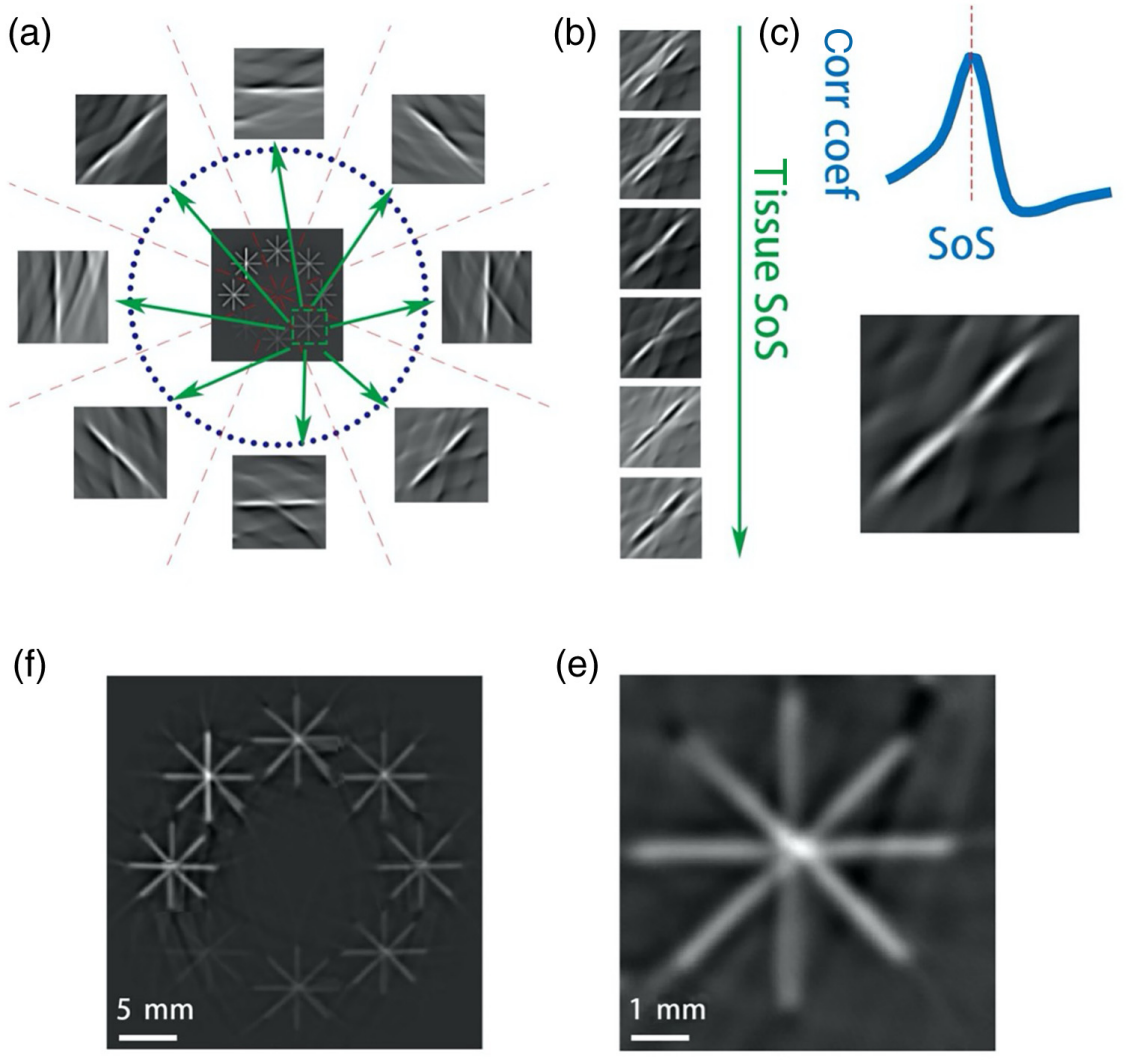
Diagram of the MSFC method. All images are obtained from numerical simulation. (a) Center: ground-truth initial pressure (IP) image. Blue dots: ultrasound transducer array positions. Outer: IP images of the region marked by the green dashed box reconstructed using signals detected by the eight sub-arrays. (b) A stack of images [of the region marked in (a)] reconstructed with increasing tissue SoS. All images are reconstructed using signals from the 4 o’clock to 10 o’clock sub-array pair. (c) Correlation coefficient between the images reconstructed using the 4 o’clock and 10 o’clock sub-arrays, plotted against the SoS. The peak determines the mean SoS along the 4 o’clock to 10 o’clock direction. (d) The image corresponding to the highest correlation coefficient. (e) The local image combining the results from all detectors. (f) The final reconstructed IP image by stitching all processed patches.

In filtered back-projection, if the distribution of SoS is given, then $\mathrm{TOF}(r^{\prime },r)$ can be readily calculated. Therefore, the reconstructed PA image is expected to have a high quality, with little feature splitting, blurring, and distortions. However, in many cases the SoS distribution is unknown and has to be estimated. In MSFC, for a ring array system, the detectors are divided into eight sub-arrays, as Fig. 1(a) shows. Eight IP images (sub-image) are reconstructed by the DAS method [keeping only the first term in the integral of Eq. (1)] separately using the data from the sub-arrays. In each sub-image, only the image components perpendicular to the line connecting the middle-points of the two opposite sub-arrays are clearly visible. Therefore, the two sub-images reconstructed from a pair of opposite sub-arrays depict the same features. If the estimated SoS is not accurate, the features on the two images will not completely match. As a result, the final image reconstructed by the full array will appear blurry. By maximizing the similarity of the two sub-images from opposite sub-arrays, the image will be in focus, and the SoS distribution can be estimated. The equation is written as^35^

$$\overset{˜}{\Lambda }=\underset{\Lambda }{\arg\;\max}[c(\Lambda )].$$ (2)

Here Λ and $\tilde{\Lambda }$ denote the real and estimated SoS distributions, respectively, and c denotes the similarity metric (usually the correlation coefficient).

During the reconstruction process, several patches with high-intensity features are chosen as the region of interest (ROI); as shown in Fig. 1(a), one of the patches is marked by the green dashed box. For each patch, eight images corresponding to the eight sub-arrays are reconstructed, as shown in Fig. 1(a). In each sub-image, the only visible features are those parallel to the corresponding sub-arrays, and images reconstructed by opposite sub-arrays depict the same features. Here, we do not need to assume that the acoustic loss is low because the MSFC method uses the correlation coefficient as the criteria, and it is immune to image intensity changes. Figure 1(b) shows a series of images by combining the reconstructed IP images of two opposite sub-arrays and adjusting the SoS. The whole tissue is considered to be homogeneous in the DAS process, whereas water is assigned a different SoS according to the temperature. Once the tissue SoS is adjusted to be equal to the mean SoS along the direction perpendicular to the main image feature, the similarity metric (correlation coefficient) between the two images reaches the global maximum, as shown in Fig. 1(c), and the combined image is shown in Fig. 1(d). After obtaining all four direction-specific SoS, the final IP image is reconstructed by linearly superimposing the four IP images reconstructed based on their direction-specific SoS, as shown in Fig. 1(e). After processing all of the image patches following the same routine, the final IP image is reconstructed by stitching all of the patches, as shown in Fig. 1(f).

After obtaining the direction-specific SoS of all regions, we can give a rough estimation of the SoS distribution. In principle, the SoS distribution can be reconstructed by back-projection, as used in ultrasound tomography. However, the information acquired is too limited to apply the back-projection method.

Therefore, we use a model-based method to reconstruct the SoS distribution. In short, several points (usually approximately 5 to 7) are picked in the tissue region where the SoS is calculated; then, the global SoS distribution is generated by natural interpolation between the discrete points. The estimation process is done by comparing the direction-specific SoS and the mean SoS computed based on the generated SoS map. A detailed description is provided in the Appendix.

Unlike ultrasound tomography, the resolution of the SoS estimation under IP-SoS joint reconstruction is relatively low.^21^^,^^34^^,^^35^ Although 5 to 7 points cannot fully characterize the SoS distribution, they can provide much useful information.^35^ Therefore, we assume that such joint reconstruction is useful for both clinical practice and laboratory studies. Ultrasound tomography has shown that, for most parts of animals, there are only a few different SoS. Therefore, we propose that approximately 5 to 7 different SoS are enough for most applications.

## 3 Results

### 3.1 Numerical Simulation

**Fig. 2:**
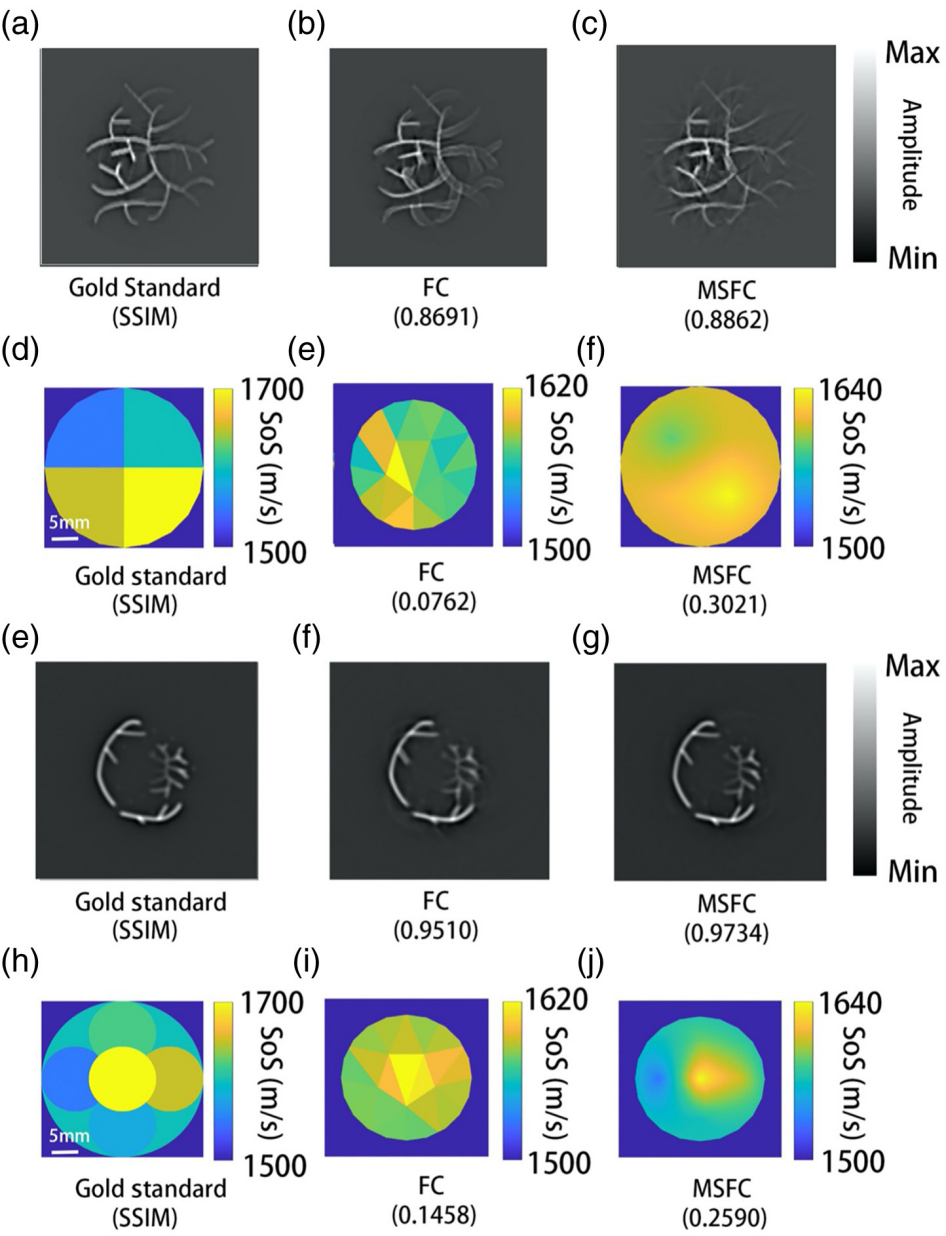
Numerical simulation results. The SSIM between each image and the gold standard is shown under each subpanel. (a), (e) Reconstructed IP image based on ground truth SoS distribution. (b), (f) Reconstruction result by the conventional FC method. (c), (g) Reconstruction result by MSFC. (d), (h) The true SoS distribution of the numerical phantom. (e), (i) SoS distribution estimated by FC. (f), (j) SoS distribution estimated by MSFC.

We used the k-Wave toolbox to generate the numerical phantoms used in this study.^36^ In the numerical simulation, the sensor size was 0.3 mm and the grid size was 0.2 mm in both directions; the directivity of the sensor was already considered. There were 512 transducers, and the radius of the ring was 5 cm, which simulated the ring array system that we used in the *in vivo* experiments. The gold-standard IP distribution is shown in Figs. 1(a) and 1(h), in the central part. The gold-standard SoS map is shown in Figs. 2(d) and 2(h), and the IP image reconstructed by single-SoS DAS is shown in Fig. 2(a) after tuning the SoS for best image quality, from which one can clearly see feature splitting due to unmatched SoS. For this numerical phantom, the signal strengths varied dramatically over space. As we discussed before, the conventional FC method is affected mostly by bright image features, while dim features are always ignored and remain blurry. As expected and shown in Figs. 2(b) and 2(f), indeed the conventional FC method failed to remove the splitting artifacts in several of the weak features. In contrast, MSFC was able to remove almost all slitting artifacts and yielded a better overall image quality, as shown in Figs. 2(c) and 2(g). Meanwhile, the FC method failed to provide a reasonable estimation of the SoS distribution. The structural similarity index measure (SSIM) between the gold standard SoS distribution and the SoS estimation by FC was as low as 0.0762/0.1458.

Once the mean SoS values along the four sampled directions are obtained, they are used to calculate the SoS distribution map. The SoS distributions calculated using FC and MSFC are shown in Figs. 2(e)/2(i) and 2(f)/2(j), respectively. Compared with the real SoS distribution shown in Fig. 2(d), the estimation by MSFC is a smoothed version of the real SoS distribution. Here, “smooth” applies to both spatial and amplitude distributions: compared with the ground truth, the estimated SoS varied in a smaller range and were spatially distributed with less abrupt changes. The reason is that more information is needed to recover finer details of the true SoS map. However, compared with the original FC method [Figs. 2(e) and 2(i)], the SoS map produced by MSFC is much better. It should be noted that, in the conventional FC method, IP and SoS are reconstructed jointly; therefore, it is difficult to adjust the local wavefront: changing the SoS locally will globally modify the wavefront. As a result, in FC, one either introduces prior knowledge about the SoS distribution, or manually divides the tissue region into many areas with a piece-wise constant SoS, and optimizes the SoS of each small area. When degrees of freedom are many, the optimization process is very complex and tends to fall into local extrema. By contrast, in MSFC, wavefront correction and SoS estimation are done sequentially, which dramatically reduces the computational cost and improves robustness. Using the MSFC method, both the reconstructed IP image and the SoS estimation are improved. Here, structural SSIM was introduced to quantify the improvement. Compared with the FC method, the new method increased the SSIM of the IP image from 0.8691 to 0.8862, whereas a more dramatic improvement from 0.0762 to 0. 3021 was observed for the SoS distribution estimation. As for another phantom, the new method increased the SSIM of the IP image from 0.9510 to 0.9734, whereas the SSIM of the SoS image improved from 0.1458 to 0.2590.

### 3.2 Phantom Studies

The SoS of agarose gel depends on the agarose concentration. Generally speaking, the SoS of agarose gel varies between 1500 and 1650  m/s, which is a little higher than water (1480  m/s at 20°C) and is similar to soft tissue. Here, a dual-SoS phantom was made with agarose gel. India ink was used to provide optical absorption, and intralipid was used to provide optical scattering. Before making the phantom, India ink was diluted to a 0.625% w/w solution. The agarose gel used here was made of 5%w/w agar, 0.5%w/w intralipid, and 0.85%w/w diluted ink. The SoS of the gel was around 1600  m/s. Inside the phantom, there was a 9 mm-diameter cylindrical cavity filled with water. Thus, the phantom was made acoustically heterogeneous, with agarose gel as a high-SoS background and water as a low-SoS inclusion. There were two vessel-like PA features near the low SoS region, drawn by 66% diluted ink. The laser used in the experiment was a 1064 nm Nd: YAG laser, pulsed with a roughly 10 ns temporal width and 10 Hz rep-rate. The laser energy density on the sample was $6\;\mathrm{mJ}/{\mathrm{cm}}^{2}/\text{pulse}$. The system applied a 256-element full-ring ultrasound detector array (Imasonic Inc.; 5.5 MHz central frequency; 60% bandwidth). Two data acquisition units (Analogic Corp., SonixDAQ; 40 MHz sampling rate; 12-bit ADC; 36 to 51 dB programmable gain) were adopted for data acquisition. To double the sampling density, the array was rotated by an angle of 2π/512 before adjacent acquisitions were made.

The water temperature was controlled to be 25.6°C, measured by a thermocouple, corresponding to an SoS of 1498.3  m/s.^10^ Before performing MSFC reconstruction, we used half-time DAS^37^ to reconstruct a raw PA image, from which the outline of the phantom was manually drawn. During the MSFC reconstruction process, the regions containing the two vessel-like PA features were chosen as the ROI.

**Fig. 3:**
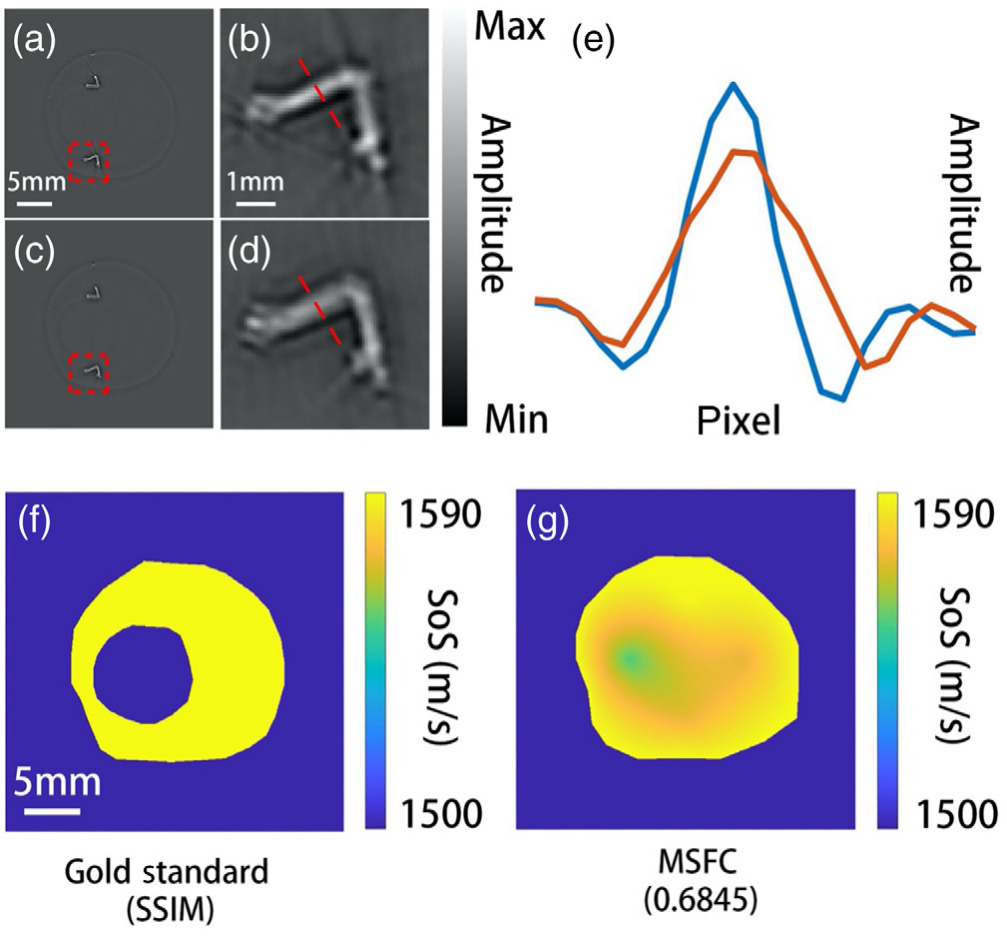
Phantom experiment results. The SSIM value between the estimated and the gold standard SoS distributions is shown. (a) IP image reconstructed by MSFC. (b) Zoomed in view of the region marked by the red box in (a). (c) IP image reconstructed by the conventional dual-SoS FC method. (d) Zoomed in view of the region marked by the red box in (c). (e) The cross-sectional profiles along the red dashed lines marked in (b) and (d), red and blue lines for conventional FC and MSFC, respectively. (f) Ground truth SoS map. (g) SoS map estimated by MSFC.

Conventional FC was also applied for comparison. In the conventional FC method, the whole phantom region (including both the water cavity and the agarose gel) was treated as acoustically homogeneous. In this case, the SoS of the phantom was set as 1585  m/s by FC. The result is shown in Fig. 3(c) [details are zoomed in and shown in Fig. 3(d)]; apparently the feature looks blurry. Thus, it is impossible to bring every feature into sharp focus given a single SoS for reconstruction. In comparison, the result produced by MSFC is sharper and clearer, as shown in Fig. 3(a) [details are zoomed in and shown in Fig. 3(b)], because the acoustic wavefront aberration was better dealt with by considering the SoS along multiple directions. The improvement of image resolution is better revealed by Fig. 3(e) in which the cross-sectional profiles along the dashed lines in Figs. 3(b) and 3(d) are co-plotted. The true (designed) SoS map is shown in Fig. 3(f), where yellow represents agarose and blue represents water. The estimated SoS distribution is shown in Fig. 3(g), which matches the ground truth reasonably well, despite the smoothing effect as predicted in our numerical study.

### 3.3 Animal Experiment

In the *in vivo* experiments, mice were maintained under gaseous anesthesia of 1.3% volatile isoflurane. Each mouse was immobilized on a lab-made animal holder upright in the center of the ultrasound detector array (the same array used in the phantom experiment). The trunk of the mouse was immersed in distilled water at around 31°C. Raw data was averaged 131 times to enhance the signal-to-noise ratio (SNR). All animal experiments were conducted in conformity with the regulations of the Laboratory Animal Research Center at Tsinghua University, Beijing, China. Mice could be imaged repeatedly without noticeable adverse conditions due to imaging. In all experiments, the excitation light had a wavelength of 1064 nm, a pulse duration of roughly 10 ns, and a repetition rate of 10 Hz. The excitation light was delivered to the animal through a 1×10 fiber bundle and illuminated the surface of the animal evenly with a surface energy density of $11\;\mathrm{mJ}/{\mathrm{cm}}^{2}/\text{pulse}$, which was below the safety limit of biological tissue.

At body temperature, coconut oil has a markedly low SoS of around 1300  m/s^38^ and remains liquid, whereas it solidifies below room temperature. We performed imaging experiments on a mouse with its stomach filled with coconut oil to create noticeable SoS inhomogeneity inside the mouse body. After the experiment, the mice were euthanized for cryotomy. Because the coconut oil had already solidified and frozen in shape, distribution of the SoS could be obtained. The experimental object was an eight-week old Crl: NU-Foxn1nu mouse. Before the experiment, the mouse was given an 8-h ambrosia to empty the stomach. The mouse was given 0.6 ml coconut oil by intragastric administration. Six layers across the stomach region were imaged sequentially at a step size of 0.28 mm. The mouse was euthanized and frozen immediately after image acquisition.

**Fig. 4:**
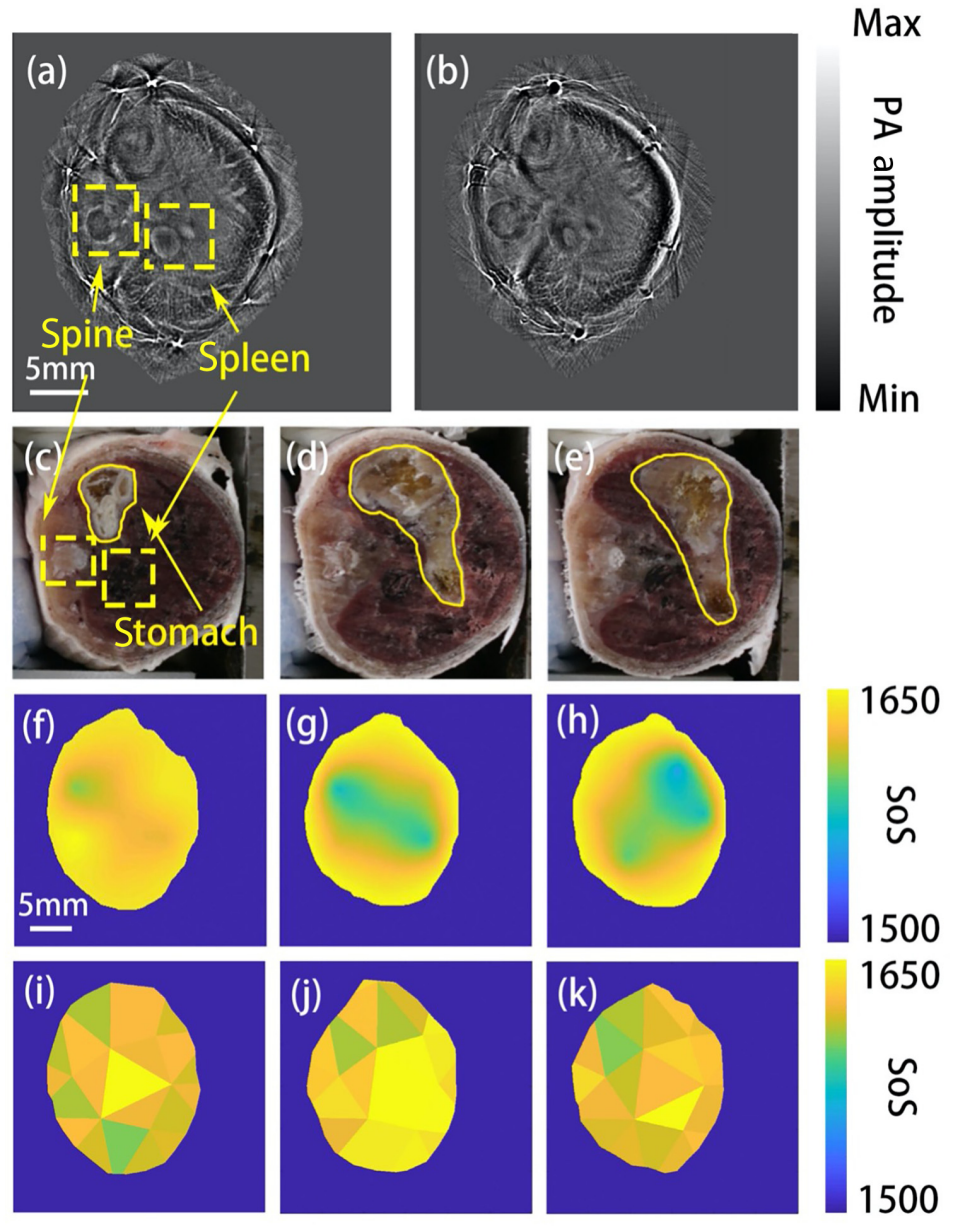
*In vivo* animal experiment results. (a) Image reconstruction result by MSFC. (b) Image reconstruction result by DAS. Both (a) and (b) correspond to the cryotomy photos of the mouse’s stomach. Spine and spleen are marked by yellow dashed line boxes, the corresponding region in the cryotomy photo. Panel (c) is also marked by a yellow dashed box. (c)–(e) The cryotomy photos of the mouse’s stomach roughly at the three imaged layers shown in (f)–(h). The white region is coconut oil, marked by the yellow solid line. (f)–(h) The estimated SoS distribution of corresponding layers. The speed of sound in the coconut oil region is lower. (i)–(k) The SoS estimation results at the same layers by the conventional FC method.

MSFC and Single-SoS DAS were applied to reconstruct the photoacoustic images; the results are show in Figs. 4(a) and 4(b), respectively. Compared with DAS, MSFC improves the image quality, with reduced feature splitting and blurring. Although there is no gold standard IP image for the animal experiment, the thin vessel near the skin may highlight the improvement of our method. If reconstructed properly, a vessel perpendicular to the imaging plane will be reconstructed into a point, as Fig. 4(a) shows. If the SoS estimation is wrong, the vessel will be distorted into a ring shape, as Fig. 4(b) shows. Therefore, we conclude that the MSFC method improved the image quality.

Cryotomy photos of the stomach region were taken, and were shown in Figs. 4(c)–4(e). The SoS distributions at the corresponding layers calculated by MSFC are shown in Figs. 4(f)–4(h), it is clear that the SoS of the stomach region (coconut oil) was significantly lower, with the profile and location roughly matching those in the cryotomy photos. In comparison, the SoS distributions estimated by the conventional FC method are shown in Figs. 4(i)–4(k), which, without prior image segmentation, failed to reveal the low SoS region. This experiment clearly demonstrates that MSFC is superior to the original version of FC when applied *in vivo*.

## 4 Discussion and Conclusion

In this paper, we demonstrate a method for IP and SoS joint reconstruction based on an advanced FC method. As for the numerical phantom, MSFC greatly improves image quality and provides satisfactory SoS estimation results. For both phantom and *in vivo* experiments in which acoustic heterogeneity exists, MSFC provides good results with reasonable time consumption. It took around 300 s in total to generate an IP image. Generating an SoS distribution map will cost another 30 s. Although it takes much longer than the popular half-time back-projection method (7 s for a full 512 ring array data), it is much shorter than the original FC method (more than 2700 s). (All algorithms were executed on Intel^®^ Core™i7-7700 CPU @ 3.6 GHz.) However, for the time being, both delineating the tissue outline and selecting the ROI were conducted manually. In the future, these procedures can be done automatically.

The speed of sound of the tissues depends on frequency, but in PACT imaging, the frequency band of the receiver is limited. As a result, the SoS can be treated as independent on the ultrasound frequency, thus the effect of acoustic dispersion is ignored. In this paper, all of the SoS estimation results represent the SoS at the ultrasound frequency around 5 MHz.

In the conventional reconstruction methods, image quality will degrade if the SoS distribution is inhomogeneous and unknown, which is always the case for *in vivo* imaging. By applying MSFC, an image with most image features in-focus can be generated even if the distribution of SoS is unknown. Meanwhile, when image features are abundant, more information regarding the acoustic wavefront distortion can be accessed, enabling MSFC to compute the SoS distribution. Here, the SoS map is directly computed after the distortions in the IP image are corrected; as a result, compared with the existing JR methods, the ill-posedness of MSFC is dramatically reduced.

**Fig. 5:**
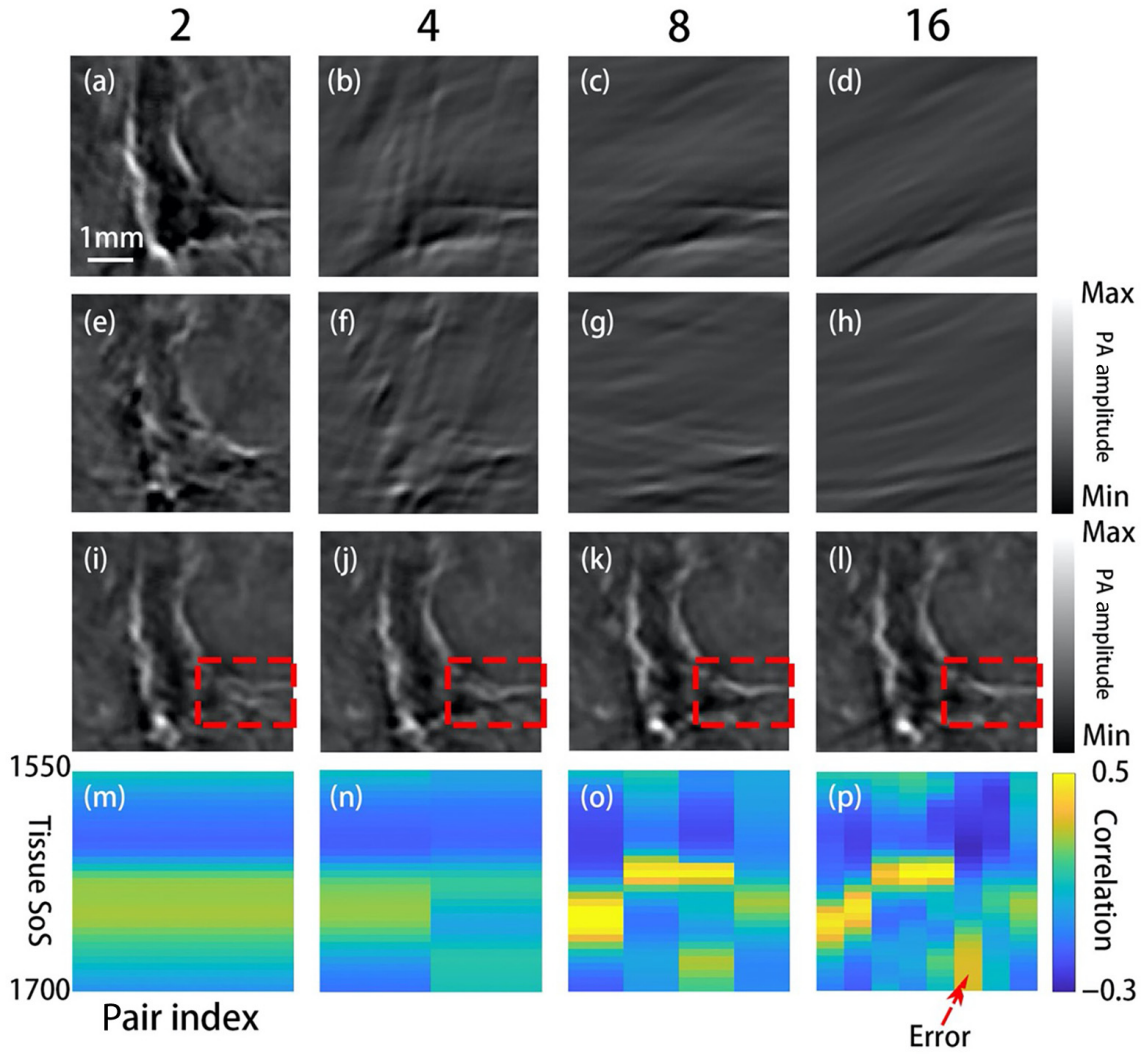
The results of different division schemes. All images are obtained from animal experiments. (a)–(d) IP images reconstructed using signals detected by 256, 128, 64, and 32 elements. (e)–(h) IP reconstruction results due to opposite elements. Panels (a)–(h) share the same color bar. (i)–(l) IP reconstruction results using different division schemes. Splitting features are highlighted by the red dashed box. (m)–(p) Correlation coefficient between the images of opposite detector groups, plotted as a function of pair index and mean SoS. The error is labeled by the red arrow.

In the original FC method, the ring array is divided into two parts, with no direction information. At the other extreme, each detector is considered to be a sub-region. The reconstruction result is generated by convolving the photoacoustic signal from each detector and its opposite detector. Our method works best for the ring array because the in-plane information in all directions is collected. In real practice, the user is free to choose the array-division scheme. To justify the current scheme of dividing the ring into eight partial arrays, we performed the following test and the results are shown in Fig. 5. The PA data were acquired from the torso of a nude mouse. We divided the detectors into 2, 4, 8, and 16 sub-arrays successively and observed the corresponding IP images and the SoS-estimation results. Figures 5(a)–5(d) show the IP reconstruction results using signals detected by half, fourth, eighth, and sixteenth of the full array, and Figs. 5(e)–5(h) show the IP images reconstructed using signals from the opposite detectors. As the number of divisions increases, the amplitude of the image becomes lower, and thus the estimation process becomes more susceptible to interference. Figures 5(i)–5(l) show the reconstructed images after superimposing all subarray images, which represent the overall quality of different division schemes. Figures 5(m)–5(p) show the correlation coefficient between image pairs as a function of SoS and index pair, plotted for all division schemes. If the number of subarrays is too many, as Figs. 5(d), 5(h), 5(l), and 5(p) show, the SNR of the reconstructed image will be too low, and thus the reconstruction is sensitive to all kinds of interference. On the other hand, if there are only two subdivisions, the precision is low, and the reconstructed IP images are blurred, as Figs. 5(a), 5(e), 5(i), and 5(m) show. The division scheme faces a trade-off between robustness and precision. For the first three division schemes, the estimation results are normal. For the last division scheme, however, the SoS estimation result of one direction (5 o’clock to 11 o’clock pair) is abnormally higher, which suggests that this division scheme produces artifacts in the SoS image. Therefore, we divided the detector ring into eight sub-arrays in all experiments.

All of the experiments here used a full-ring PACT system. With moderate modification, the same idea can be extended to work with other array geometries, except for the linear array or arc array because there are no “opposite detectors” in linear arrays or arc arrays. We tested the new algorithm on a columnar array for breast imaging and may report the result in the future. In sum, we envision that dividing the detectors array into several sub-arrays and maximizing the correlation coefficient among their respective images can improve image quality by removing distortions and splitting artifacts. As a bonus, by utilizing the mean SoS along different directions, the global SoS distribution with an accuracy that is dependent on the abundance of the fine features in the IP image can be estimated.

## 5 Appendix A

Pseudo code of multi-segmented feature-coupling (Table 1).

**Table 1:** Multi-segmented feature-coupling method.

**Set parameters**
Draw the outline of the animal trunk/phantom based on an image reconstructed by half-time back-projection. Set an SOS range, for example, approximately 1600 to 1700 m/s for animal experiments. At an increment of 5 m/s, scan the SoS of the reconstruction algorithm and reconstruct the IP image by DAS, using data from one subarray. Save the results. Repeat the above for all eight subarrays. Thus, we finally save 168 (21×8) images.
Run MSFC
1. Manually choose a region of interest.
2. Choose a pair of detector groups.
3. Calculate the correlation coefficient between images of opposite groups under different SoS, and find the maximum.
4. Go to step 2, and choose another pair of detector groups.
5. Go to step 1, and choose another region of interest.

## 6 Appendix B

Pseudo code of SoS estimation (Table 2).

**Table 2:** SoS estimation method

**Set parameters**
After reconstructing the IP image, the mean SoS along the ultrasound propagation path is known. The information provided by the mean SoS is not sufficient for back-projection based SoS estimation, so we use a model-based method to estimate the SoS distribution.
Set n (4<n<7, empirically) points inside the phantom/animal trunk region. The SoS of these points is $\Lambda =(v_{1},v_{2},\ldots ,v_{n})$, and generate the final SoS distribution map by natural interpolation.
An initial SoS $v_{0}$ could be chosen using other SoS compensation methods. Also, set the upper limit of the iteration number N, the iteration step τ, and the SoS perturbation Δv. Set the initial iteration number k=1.
**Run SOS estimation**
1. Given the SoS of several points $\Lambda_{k-1}$, and calculate the SoS distribution map Ω($\Lambda_{k-1}$) by natural interpolation.
2. Calculate the mean SoS along the ultrasound propagation path, mathematically $M(\Omega (\Lambda ))=({\overset{¯}{v}}_{1},{\overset{¯}{v}}_{2},{\overset{¯}{v}}_{3},\ldots ,{\overset{¯}{v}}_{m})$.
3. Calculate the correlation coefficient between M(Ω(Λ)) and the mean SoS derived from the MSFC method; the correlation is written as $c(\Lambda_{k-1})$.
4. Add a perturbation Δv to $\Lambda_{k-1}$, and calculate $c(\Lambda_{k-1}+\Delta v)$ as described in 1, 2, and 3.
5. Calculate the gradient correlation coefficient $\nabla c(\Lambda_{k-1})=c(\Lambda_{k-1}+\Delta v)-c(\Lambda_{k-1})$.
6. Compute the increment $\mu =\tau \nabla c(\Lambda_{k-1})$.
7. $\Lambda_{k}=\Lambda_{k-1}+\mu$
8. k=k+1.
9. if k<N, go to step 1; otherwise output the result Λ.

## Code, Data, and Materials Availability

Code and data will be provided upon request.
